# Rectal Atresia Treated Via a Transanal and Posterior Sagittal Approach: A Report of Two Cases

**DOI:** 10.7759/cureus.38694

**Published:** 2023-05-08

**Authors:** Maho Kurashima, Samrudhi Joshi, Justin Sobrino, Christopher Blewett

**Affiliations:** 1 Pediatric Surgery, SSM Health Cardinal Glennon Children’s Hospital, Saint Louis, USA; 2 Pediatric Surgery, Saint Louis University School of Medicine, Saint Louis, USA

**Keywords:** neonatal surgery, posterior saggital anorectoplasty, neonatal bowel obstruction, anorectal malformation, rectal atresia

## Abstract

Rectal atresia is a rare cause of bowel obstruction in neonates with a normal-appearing anus. We present two different types of rectal atresia requiring different surgical management. Case one was a one-day-old term male with web-type rectal atresia diagnosed preoperatively with bedside obliteration of the web. Subsequent transanal web resection was performed. Case two was a one-day-old male born at 28 weeks weighing 980 g with significant cardiac defects including aortic atresia. The patient underwent initial colostomy creation and delayed rectal anastomosis via posterior sagittal anorectoplasty. We review the published literature, discuss the surgical strategy, and highlight the decision-making of diverting ostomy creation and approach of definitive anorectal anastomosis.

## Introduction

Rectal atresia is a rare but possible cause of large bowel obstruction in neonates with a normal-appearing anus. This anorectal malformation, in contrast to others, involves a patent anal orifice at the normal location and normal development of the internal and external anal sphincters [1,2]. Most reported cases had no fistula between the rectum and the urogenital tract. Previous studies have described different surgical strategies, including initial diverting ostomy creation with delayed definitive surgery versus primary repair [3-11]. However, due to the rarity and wide variety of diseases, there is no absolute surgical strategy for preoperative diagnosis and decision-making. Regarding the surgical approach for definite correction, various approaches, including posterior sagittal anorectoplasty (PSARP), transanal pull-through, and magnetic compression anastomosis, have been reported [3,7,11-13]. However, a small number of case series and reports describe the long-term outcome after repair. Considering the normal function and anatomy of the anal sphincter, a surgical approach accompanied by dissection of the perineal muscles should be avoided as much as possible. In this report, we present two distinct rectal atresia cases. One of them is a web-type rectal atresia that was successfully diagnosed preoperatively and treated with web resection. The other is rectal atresia with an atretic chord type requiring initial diversion and delayed anorectal anastomosis via PSARP.

## Case presentation

Case one

**Figure 1:**
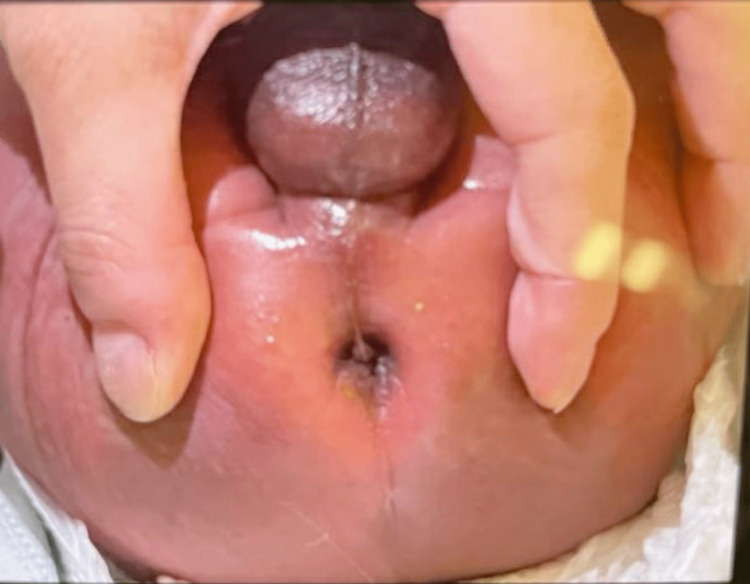
Physical examination of the anus. The anus appeared in the normal position with matured gluteal muscle and skin wrinkles.

A one-day-old male was born at term weighing 3.1 kg. His prenatal history was unremarkable, and delivery was uncomplicated. He had not passed stool since birth and had increased bilious emesis and abdominal distention. The anus appeared in the normal position with matured gluteal muscle and skin wrinkles, as seen in Figure 1.

**Figure 2:**
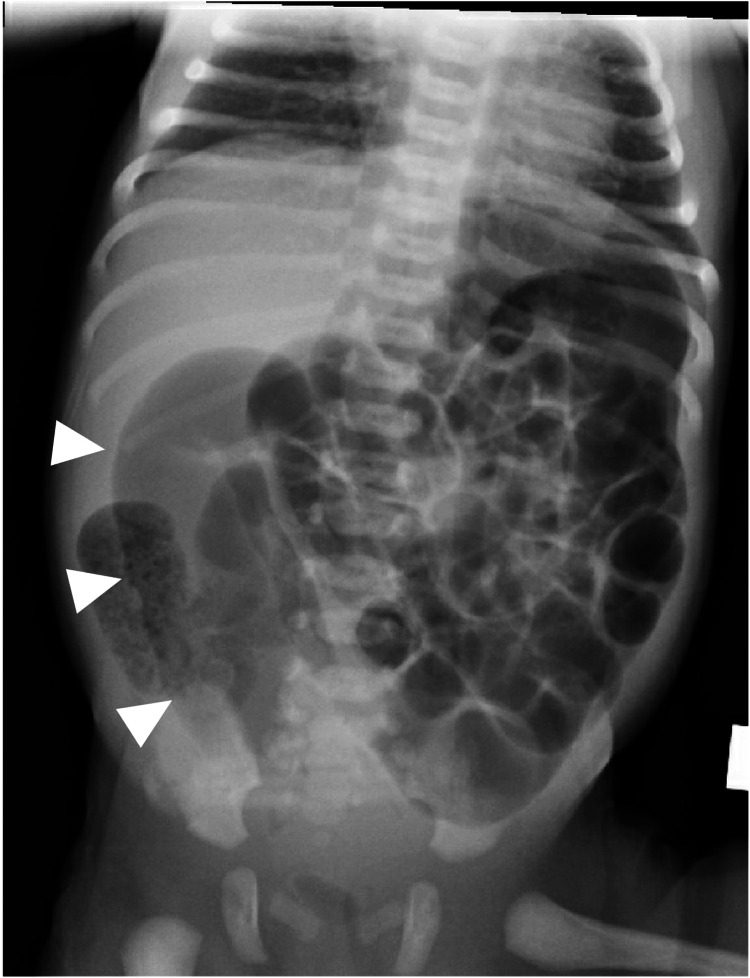
Abdominal X-ray. Multiple dilated bowel loops with an S-shaped dilated loop of bowel traveling downward toward the pelvis representing the sigmoid colon (white arrowheads).

His genital examination demonstrated well-formed, normal-appearing male genitalia without obvious evidence of a fistula. His urine was clean without contamination from meconium. No catheter traveled more than half an inch through the anal orifice. The echocardiogram, renal ultrasound, and vertebral X-ray were normal without evidence of VACTERL association. The abdominal X-ray depicted multiple dilated bowel loops with an S-shaped dilated loop of bowel traveling downward toward the pelvis representing the sigmoid colon, as depicted in Figure 2.

**Figure 3:**
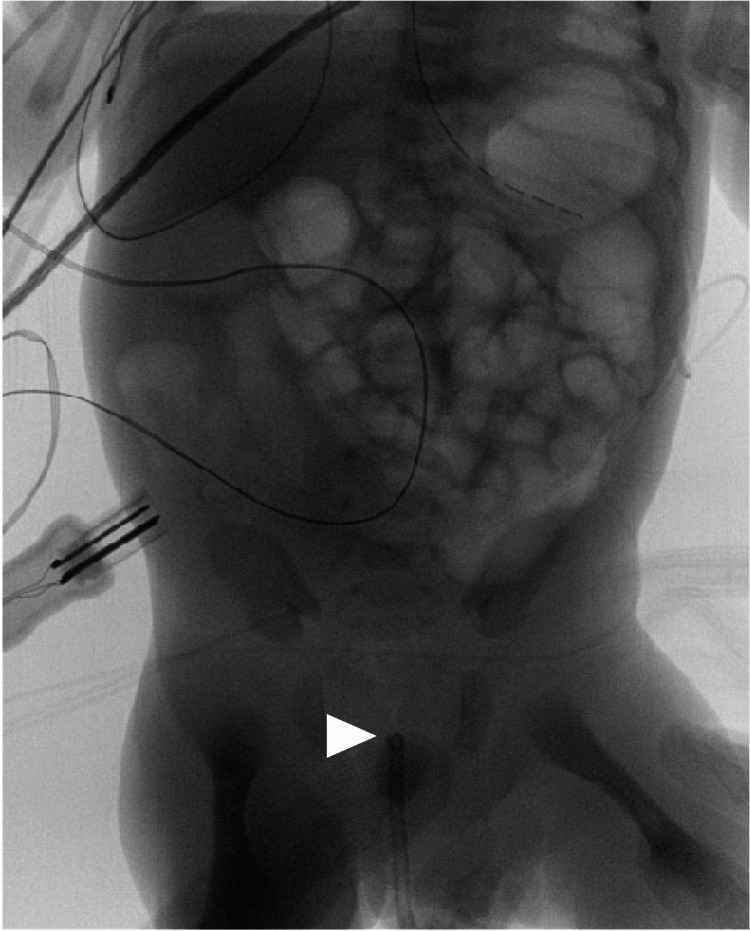
Lower gastrointestinal fluoroscopy. The contrast did not pass through the rectum. The white arrow shows where the tip of the catheter met resistance.

Lower gastrointestinal fluoroscopy showed no contrast passage through the rectum, and the catheter tip met resistance at half an inch from the anal orifice, as seen in Figure 3.

The physical examination and image findings were consistent with rectal atresia. After the diagnosis was confirmed, we gently inserted an 8-Fr Salem sump tube through the anal orifice. The tube was pushed through with some resistance, and the meconium started to pass. We suspected that we obliterated a mucosal web, which further solidified the decision to perform an examination under anesthesia in the operating room on the day of life (DOL) five. Operative findings showed that only a lacrimal probe was able to pass through the orifice initially. During a more detailed inspection, a posteriorly placed epithelial band that was partially obstructing the anal opening was identified. The diagnosis of rectal atresia type 1b with a mucosal web was confirmed. The band was divided using electrocautery. Then, the anal orifice was dilated using Hegar dilators, achieving a maximum dilation of up to size 10. Postoperatively, serial anal dilations were continued daily to prevent further adhesion. The patient has been stooling and micturating for one year postoperatively.

Case two

**Figure 4:**
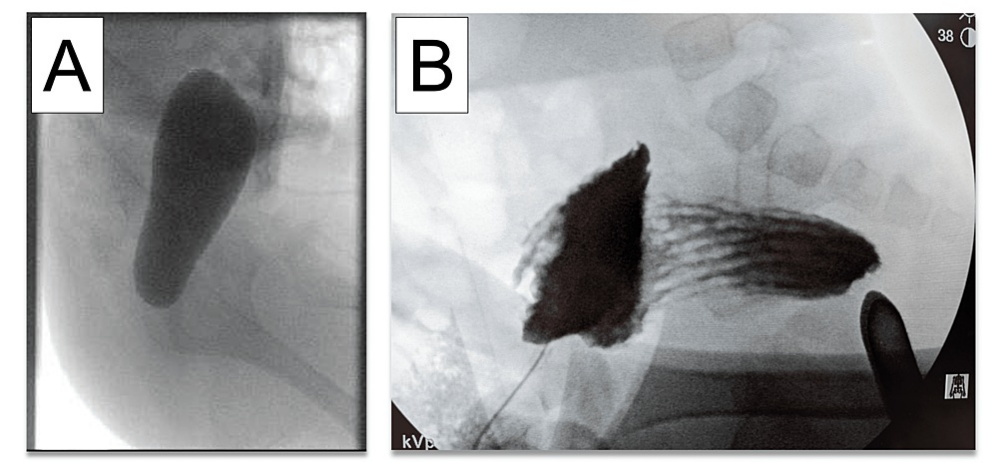
Distal colostogram. A. Perioperative colostogram showed a blind-ended rectum without any evidence of a fistula. B. Intraoperative distal colostogram with Hegar dilator inserted via the anal canal showed about 1 cm of distance between the proximal and distal rectum pouch.

A one-day-old male was born at 28 weeks and four days of gestation weighing 980 g by emergency C-section due to multiple gestations, breech presentation, and premature rupture of membranes at an outside hospital. The patient was prenatally diagnosed with congenital heart disease, including aortic atresia. Meconium had not passed since the birth, and abdominal distention was noted on DOL one. The anus was located in the normal position. A feeding tube inserted in the anal canal met resistance at approximately 1.5 cm from the anal opening without passage of the meconium. His abdominal X-ray was relevant for diffuse intestinal and colonic dilation. The patient was diagnosed with rectal atresia, and a sigmoid colostomy was created on DOL one. Postoperatively, the patient was transferred to our hospital for management of his comorbidities, including chronic bronchopulmonary dysplasia with a tracheostomy and congenital cardiac disease including ventricular septal defect, aortic atresia with aortic arch hypoplasia, and hypoplastic left heart syndrome status. After Rastelli’s surgery was completed, a definitive repair of rectal atresia was planned at the age of 10 months when the patient weighed 8.4 kg. His preoperative distal colostogram showed a blind-ended rectum without any evidence of a fistula, as seen in Figure 4.

**Figure 5:**
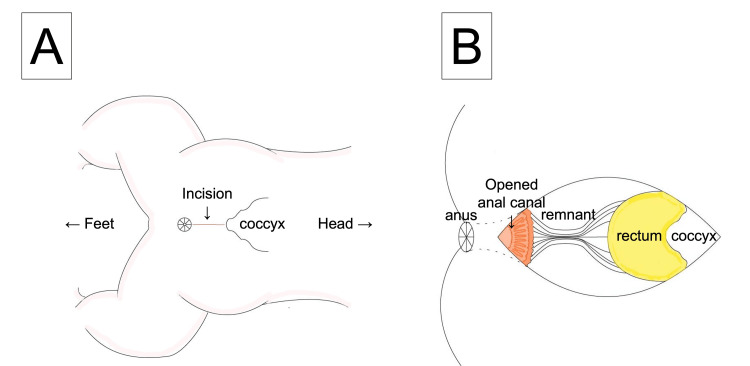
Operative findings. A. The incision for posterior sagittal anorectoplasty. B. An atretic chord measuring several centimeters between the blind end of the anal canal and the blind pouch of the rectum. The anterior wall of the blind end of the anal canal was opened.

We performed an intraoperative distal colostogram, which demonstrated that the length of the atretic part of the rectum could be approached via a posterior sagittal incision. The decision was made to proceed with PSARP. Operative findings showed an atretic chord measuring several centimeters between the blind end of the anal canal and the blind pouch of the rectum, as described in Figure 5.

No fistula was noted between the rectum and urethra. The diagnosis of rectal atresia type III with an intervening fibrous cord was confirmed. The proximal rectal pouch was dissected several centimeters proximally, and the rectal pouch was opened. The anal pouch was opened vertically at the posterior wall and anastomosed circumferentially with the rectum using 4-0 Vicryl interrupted stitches while reconstructing the anal canal, as shown in Figure 5. Hegar number 8 was passed easily through the anastomosis. The patient remained diverted at that time, and his ostomy was reversed two months later. The patient has had no reports of constipation with normal urine and stool output at three months postoperatively.

## Discussion

**Figure 6:**
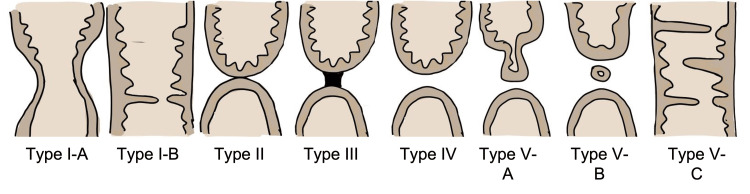
Different types of rectal atresia. Different types of rectal atresia, as described by Sharma and Gupta [15]. Reproduced with permission from the author. Case one is type IB, and case two is type III.

Rectal atresia has a reported incidence of 0.3-1.2% of all anorectal malformations [14]. It shows marked male predominance with a male-to-female ratio of 7:3 [14]. Unlike other types of anorectal malformation, rectal atresia is characterized by normal anal position, well-developed pelvic structures, and functional external and internal sphincters [15]. Rectal atresia is further classified into different types according to Sharma and Gupta. A reproduced diagram from Sharma and Gupta is shown in Figure 6 [15].

Most rectal atresia cases have no associated fistula with the urogenital system [14]; however, some studies reported rectal atresia being associated with rectovestibular fistula [16,17]. Various notable medical disorders were discussed in the case series that were concurrent with rectal atresia, including sacral masses, double vagina and uterus, cardiac anomalies such as patent ductus arteriosus and ventricular septal defect, and congenital renal anomalies such as dysplastic kidney, ectopic kidney, and duplex ureters [18,19]. Our two cases showed different clinical presentations, comorbidities, and types and were treated with distinct surgical strategies. Case one was a one-day-old full-term male with no other comorbidities who was diagnosed with rectal atresia type IB, web-type atresia. We performed transanal epithelial band resection after confirmation of the passage of meconium via bedside subversion of the web. Case two was a one-day-old, premature, extremely low-birth-weight male with significant cardiac defects. We initially performed diverting ostomy creation, and after cardiac surgery was completed, anorectal anastomosis via PSARP was performed at 10 months of age. He was classified as type III atresia with an intervening fibrous cord based on the operative findings.

The published literature was reviewed with a focus on surgical management. All cases underwent surgical intervention. In most case series, initial diverting colostomy creation was performed [11], and some case reports presented cases of primary repair without diversion [2,10,15]. These primary repair cases included magnetic compression anastomosis, web resection, and PSARP. Hence, web type can potentially be treated with only web resection, as in case one. We recommend a physical examination at the bedside with cautious insertion of a catheter for potential breakage of the web. We can avoid ostomy creation and complications that present with more invasive surgery accompanied by anastomosis. If this examination fails, the next approach should be tailored to each patient’s status. Although a distal colostogram can be useful to rule out a fistula between the urogenital tract, the necessity of the creation of a colostomy is still controversial based on the fact that a low percentage of rectal atresia cases is associated with a fistula. If the patient has a significant cardiac defect that potentially affects perfusion of the anastomosis and wound healing, initial diversion and delayed definitive surgery are preferred strategies. Neurological and developmental prognosis, which affects defecation function, should be considered as well. We chose initial diversion followed by definitive surgery for case two due to risk factors of prematurity and cardiac defects.

There was a notable variance in definitive surgery reported. Most selected procedures included anastomosis, such as PSARP, transanal pull-through, magnetic anastomosis [10], and transanal end-to-end rectoanal anastomosis [11]. Over half of the patients underwent staged repair by initial diversion and delayed reconstruction. The most common complication related to the surgeries, specifically the pull-through technique, was constipation. In one of the discussed case series, patients who did not undergo an ostomy had complications after surgery, such as presacral mass and rectovaginal fistula. There is no study comparing the complication rates in each of these approaches. In theory, rectal atresia has an intact anal sphincter, thus preserving anatomical structure without manipulation is the ideal approach. The clinical question should be: how can we decide on a surgical approach of transanal versus PSARP versus pull-through preoperatively or intraoperatively? In our case one, we diagnosed web-type preoperatively, which enabled straightforward decision-making of web resection without ostomy creation. The necessity of web resection for patients who continuously pass stool after bedside procedure and dilation remains unclear. We decided to resect the web to avoid possible future strictures due to re-adhesion, as described by Braiek et al. [20].

With the exception of web-type rectal atresias, some procedures accompanied by anastomosis will be required for the other types. If a patient undergoes initial ostomy creation, a distal colostogram will tell us the distance between the proximal rectum and the distal rectal pouch and the existence of the fistula. Radiopaque material such as a Hegar dilator inserted from the anal canal is also helpful. In case two, the intraoperative colostogram showed that the proximal rectum pouch was considered amenable to be approached by PSARP. End-to-end anastomosis was achieved under minimum tension without abdominal dissection. If the colostogram had shown a significant distance between the pouches, we would have changed our approach to a laparoscopic intra-abdominal dissection combined with PSARP/transanal pull-through with resection as the more suitable option. No study has compared PSARP with transanal pull-through for rectal atresia. Shehata et al. reported the advantage of the transanal end-to-end rectoanal anastomosis approach over the PSARP as anatomical anorectal reconstruction under direct visual control is favorable rather than an extensive anorectal dissection or any division of the sphincter musculature with simultaneous closure of the ostomy. However, to achieve tension-free anastomosis, a previous sigmoid colostomy should be positioned properly low. In case two, the ostomy was created at a relatively high position in the sigmoid colon by an outside hospital; therefore, we were inclined to proceed with PSARP rather than the transanal approach. To compare each approach, a longer period of follow-up with a higher number of cases is essential.

## Conclusions

In this report, we describe two different types of rectal atresia requiring distinct operative approaches. Rectal atresia is a rare cause of bowel obstruction in neonates with a normal-looking anal opening. There is a wide range of surgical strategies, including primary repair versus initial ostomy creation, transanal approach versus PSARP, and delayed reconstruction, which can be tailored based on the distance of the rectal pouches and other comorbid conditions.
